# Acute and chronic systemic CB_1_ cannabinoid receptor blockade improves blood pressure regulation and metabolic profile in hypertensive (mRen2)27 rats

**DOI:** 10.14814/phy2.12108

**Published:** 2014-08-28

**Authors:** Chris L. Schaich, Hossam A. Shaltout, K. Bridget Brosnihan, Allyn C. Howlett, Debra I. Diz

**Affiliations:** 1 Department of Physiology and Pharmacology, Wake Forest School of Medicine, Winston‐Salem, North Carolina; 2 Hypertension & Vascular Research Center, Wake Forest School of Medicine, Winston‐Salem, North Carolina; 3 Department of Obstetrics & Gynecology, Wake Forest School of Medicine, Winston‐Salem, North Carolina

**Keywords:** Baroreflex, endocannabinoid system, hypertension, metabolic syndrome, renin‐angiotensin system

## Abstract

We investigated acute and chronic effects of CB_1_ cannabinoid receptor blockade in renin‐angiotensin system‐dependent hypertension using rimonabant (SR141716A), an orally active antagonist with central and peripheral actions. In transgenic (mRen2)27 rats, a model of angiotensin II‐dependent hypertension with increased body mass and insulin resistance, acute systemic blockade of CB_1_ receptors significantly reduced blood pressure within 90 min but had no effect in Sprague‐Dawley rats. No changes in metabolic hormones occurred with the acute treatment. During chronic CB_1_ receptor blockade, (mRen2)27 rats received daily oral administration of SR141716A (10 mg/kg/day) for 28 days. Systolic blood pressure was significantly reduced within 24 h, and at Day 21 of treatment values were 173 mmHg in vehicle versus 149 mmHg in drug‐treated rats (*P* < 0.01). This accompanied lower cumulative weight gain (22 vs. 42 g vehicle; *P* < 0.001), fat mass (2.0 vs. 2.9% of body weight; *P* < 0.05), and serum leptin (2.8 vs. 6.0 ng/mL; *P* < 0.05) and insulin (1.0 vs. 1.9 ng/mL; *P* < 0.01), following an initial transient decrease in food consumption. Conscious hemodynamic recordings indicate twofold increases occurred in spontaneous baroreflex sensitivity (*P* < 0.05) and heart rate variability (*P* < 0.01), measures of cardiac vagal tone. The beneficial actions of CB_1_ receptor blockade in (mRen2)27 rats support the interpretation that an upregulated endocannabinoid system contributes to hypertension and impaired autonomic function in this angiotensin II‐dependent model. We conclude that systemic CB_1_ receptor blockade may be an effective therapy for angiotensin II‐dependent hypertension and associated metabolic syndrome.

## Abstract (precis)

Acute and chronic systemic CB_1_ cannabinoid receptor blockade significantly lowers blood pressure in Angiotensin II‐dependent hypertensive (mRen2)27 rats, with a concomitant positive influence over conscious autonomic blood pressure regulation and metabolic profile. Results from our study indicate novel mechanisms for maintenance of hypertension, metabolic syndrome, and impaired autonomic control of blood pressure associated with upregulation of Angiotensin II signaling.

## Introduction

Hypertension, diagnosed when arterial pressure (AP) exceeds 140/90 mmHg, is the single most important risk factor for the development of cardiovascular disease and currently affects one in three U.S. adults (Go et al. 2014). Although the mechanisms involved in the development of hypertension are still not fully understood, the widespread use of medications that inhibit the formation of the peptide hormone angiotensin (Ang) II or its actions at the ubiquitously expressed Ang II type 1 (AT_1_) receptor as first‐line treatments implicates disturbances in the renin‐angiotensin system (RAS) in the maintenance of the disease (Herichova and Szantoova 2013). RAS blockers have a beneficial profile in hypertension associated with metabolic syndrome, unlike many antihypertensive drugs that have reduced efficacy in obese or diabetic patients (Wenzel et al. 2013) or produce undesirable metabolic side effects (Lamont 1995). However, there is need for further research into the mechanisms linking the RAS with the metabolic syndrome in hypertension.

The central and peripheral endocannabinoid system has emerged as a target for the treatment of conditions associated with the metabolic syndrome, which include the cluster of risk factors for cardiovascular disease, obesity, and insulin resistance (Grundy et al. 2005). The endocannabinoid system comprises a spectrum of fatty acids, including the endogenous cannabinoids anandamide and 2‐arachidonoylglycerol (2‐AG), which exert their paracrine‐mediating effects through the widely expressed G protein‐coupled CB_1_ and CB_2_ receptor subtypes (Pertwee et al. 2010). Systemic blockade of CB_1_ receptors by selective, brain‐penetrating antagonists such as rimonabant (SR141716A) confers beneficial antiobesity and antidiabetic effects in humans and animals with metabolic syndrome [see Kirilly et al. (2012) for review], implicating overactivity of the endocannabinoid system in the pathogenesis or maintenance of these conditions. The precise role of the endocannabinoid system in promoting cardiovascular diseases associated with the metabolic syndrome, including hypertension, is less clear in part due to the inability to differentiate direct effects on AP from indirect effects mediated by weight loss or normalization of metabolic factors such as insulin or leptin sensitivity (Grassi et al. 2008; Ruilope et al. 2008). In obese Zucker rats, long‐term systemic treatment with a CB_1_ receptor antagonist normalized the pressor effect of acutely administered Ang II (Janiak et al. 2007). However, acute CB_1_ receptor blockade in lean spontaneously hypertensive rats (SHR) elevated pressure (Batkai et al. 2004), whereas chronic CB_1_ receptor antagonist treatment did not alter pressure or heart rate in obesity prone‐SHR (Slavic et al. 2013).

Evidence for signaling interactions between the endocannabinoid system and the pathogenic actions of Ang II is mounting. For example, Ang II transactivates CB_1_ receptors by stimulating production of 2‐AG in cell culture (Turu et al. 2009) and in rat arteriole beds (Szekeres et al. 2012). While in the acute setting, the 2‐AG release appears to offset the vasoconstrictor actions of the peptide (Szekeres et al. 2012), blockade of CB_1_ receptors in Sprague‐Dawley (SD) rats exposed to chronic ethanol treatment prevented Ang II‐mediated mitogenic signaling and profibrogenic gene expression in hepatic stellate cells (Rozenfeld et al. 2011). Transgenic (mRen2)27 rats, a monogenetic model of Ang II‐dependent hypertension in which the mouse *Ren2* renin gene was transfected into the genome of the SD rat, have a phenotype of chronic hypertension with markedly impaired baroreflex control over heart rate (HR), increased body weight, and reduced insulin and leptin sensitivity by 16 weeks of age compared to their normotensive genetic controls (Bader et al. 1992; Kasper et al. 2005; Sloniger et al. 2005a,b). The (mRen2)27 rat is therefore an ideal model in which to study the contribution of the endocannabinoid system to both cardiovascular and metabolic dysfunction in an in vivo setting of Ang II‐mediated pathologies. On the basis of these prior reports (Turu et al. 2009; Rozenfeld et al. 2011), we hypothesized that chronic systemic blockade of CB_1_ receptors by SR141716A would improve cardiometabolic function in this strain, possibly by disrupting the interactions between the endocannabinoid system and RAS.

## Methods

### Animals

All experiments were performed in male 15‐ to 20‐week‐old hypertensive hemizygous (mRen2)27 rats or normotensive SD rats obtained from the Hypertension & Vascular Research Center colony at Wake Forest School of Medicine. Animals were housed two per cage in a humidity‐ and temperature‐controlled room with free access to standard chow (ProLab, PMI Nutrition International, Brentwood, MO) and water. The colony maintained a 12‐h light/dark cycle (lights on at 0600). All experimental procedures were approved by the Institutional Animal Care and Use Committee.

### Experimental protocol

Acute experiments were performed in (mRen2)27 or SD rats aged 18 to 20 weeks. Animals were trained for per os (p.o.) daily oral gavage treatment with the vehicle (0.1% Tween‐80 in double‐distilled water) for seven days prior to testing, and acclimated to metabolic cages (Allentown Caging Equipment, Allentown, NJ) and the tail cuff blood pressure monitoring system (NIBP‐8, Columbus Instruments, Columbus, OH) 48 h prior to testing. On the testing day, baseline systolic blood pressure (SBP) and HR (in beats per minute [bpm]) were measured by tail cuff and recorded as the average of a minimum of 10 individual readings over a period of approximately 15 min per animal. Animals were then treated with either SR141716A (10 mg/kg p.o.; obtained from RTI International [Durham, NC] via the National Institute on Drug Abuse; *n* = 5 per strain) or its vehicle (*n* = 5 per strain). After 90 min SBP and HR were reassessed in the same manner. Animals were then placed individually in metabolic cages overnight. Rats treated with SR141716A had ad libitum access to food and water, while rats treated with vehicle were food restricted (FR) to 13 g of food, based on previous observations of the acute hypophagic effect of SR141716A in both strains in our preliminary studies and by others (Ravinet Trillou et al. 2003), to control for differences in food consumption that may contribute to acute differences in SBP (Gradin and Persson 1990). Twenty‐four hours after dosing, SBP and HR were again measured. Animals were then sacrificed by decapitation and trunk blood collected for analysis of RAS components, insulin and leptin levels. All SBP recordings were obtained between 1300 and 1600.

Chronic experiments were performed in separate groups of (mRen2)27 rats beginning at 15 weeks of age. As in our acute studies, animals were trained for p.o. injections with daily administration of the vehicle for 7 days and were acclimated to the tail cuff apparatus and metabolic cages 48 h prior to the commencement of testing. Baseline values for SBP, HR, and food and water consumption were obtained on Day 0 of the study, when animals were exactly 16‐week old. Daily oral treatment with SR141716A (10 mg/kg/day; *n* = 7) or vehicle (*n* = 8) began on Day 1 and continued for 28 days. Body weight was recorded daily through Day 25, while SBP, HR, food, and water consumption, urine volume and blood glucose were measured on treatment Days 7, 14, and 21. Overnight food and water intake and SBP were measured in subgroups of SR141716A‐treated (*n* = 3) and vehicle‐treated (*n* = 4) animals on Day 2 to assess acute feeding effects of systemic CB_1_ receptor blockade. Blood glucose measurements were taken with a Freestyle glucose meter (Abbott Diabetes Care Inc., Alameda, CA) from a ~10 *μ*L blood sample obtained from tail pricks at least 60 min after tail cuff recordings, between 1500 and 1700. Food was not withheld during this window. Urine was flash‐frozen over dry ice on Days 0 and 21 and collected for analysis of osmolality and vasopressin content. On Day 25, animals were surgically instrumented with a femoral artery catheter to record conscious hemodynamic measures including baroreflex sensitivity (BRS) for control of HR, HR variability (HRV), and blood pressure variability (BPV) on Day 28. After testing was completed on Day 28, animals were sacrificed by decapitation and trunk plasma and serum collected for analysis of RAS components, insulin, and leptin levels. Fat mass index was calculated from white adipose tissue collected from retroperitoneal, inguinal and epididymal stores and weighed as a percentage of total body weight. As in our acute studies, all tail cuff SBP, HR, and conscious hemodynamic recordings were obtained between 1300 and 1600 and approximately 90 min after dosing.

### Surgical procedures and conscious hemodynamic measures

As described previously (Shaltout and Abdel‐Rahman 2005), rats were anesthetized under 2.5 to 4.0% isofluorane and instrumented with a femoral artery catheter on the afternoon of Day 25 of the study. Rats were allowed two days to recover during which they were gavaged with water to prevent dehydration in addition to receiving daily drug or vehicle treatment. Pulsatile pressure in conscious rats was acquired on Day 28 between 1300 and 1500 via strain gauge transducer connecting the arterial catheter to a data acquisition system (Acq*Knowledge* software version 3.8.1, BIOPAC Systems, Goleta, CA). HR was calculated from the AP wave. Indices of sympathovagal activity were calculated by spectral analysis of the time and frequency domains using software designed for rats (Nevrokard SA‐BRS, Medistar, Houston, TX), as previously described (Shaltout and Abdel‐Rahman 2005). Consistent with the duration of recordings used in previous human and rodent studies (Shaltout and Abdel‐Rahman 2005; Fisher et al. 2009), conscious spontaneous BRS was determined from a minimum of 10 min of AP recordings obtained within 90 min of SR141716A or vehicle dosing. Conscious spontaneous BRS was calculated in the time (Sequence [Seq] Up, Seq Down, and Seq All; in units of milliseconds per mmHg) and frequency (low‐frequency [LF] and high‐frequency [HF] *α* indices) domains. Time‐domain analysis was used to calculate differences in HRV, an index of cardiac vagal tone, measured as the standard deviation of the beat‐to‐beat interval (SDRR) in milliseconds. BPV, an index of vascular sympathetic tone, was measured in the time domain as the standard deviation of the mean AP (SDMAP) in mmHg. Power of systolic AP (SAP) spectra was calculated as LF‐SAP for an additional measure of sympathetic tone.

### Biochemical measurements in plasma, serum, and urine

Plasma angiotensin peptides [Ang I, Ang II, Ang‐(1–7)] were measured as previously reported (Chappell et al. 2003). Serum angiotensin converting enzyme (ACE), insulin, leptin, and urine vasopressin were measured using radioimmunoassays specific for rats according to the manufacturer's instructions (Linco, Santa Fe Springs, CA) (Kasper et al. 2005). Urine osmolality was measured using the 5004 Micro‐Osmette osmometer (Precision Systems, Natick, MA) and expressed as milliOsmols per liter (mOsm/L).

### Analysis of data

Values are presented as mean ± SEM. Comparisons of changes in body weight, SBP, HR, food and water consumption, and blood glucose over time between drug and vehicle groups were analyzed by repeated measures two‐way ANOVA, with Bonferroni post hoc comparisons made between drug and vehicle groups where appropriate. Within group comparisons were made by repeated measures one‐way ANOVA, with post hoc Tukey tests used to elucidate further comparisons between timepoints. Student's unpaired two‐tailed t‐tests were used to analyze comparisons of fat mass index, biochemical measurements, conscious hemodynamic parameters on Day 28, and differences in food and water intake, SBP, and HR after Day 1 of chronic treatment in subgroups. Paired two‐tailed t‐tests were used to compare 24‐h changes in body weight from baseline in acute studies. The criterion for statistical significance was *P* < 0.05. Statistical tests were performed using Prism 5.0 (GraphPad Software, San Diego, CA).

## Results

### Acute systemic CB_1_ receptor blockade in (mRen2)27 and SD rats

**Figure 1.**
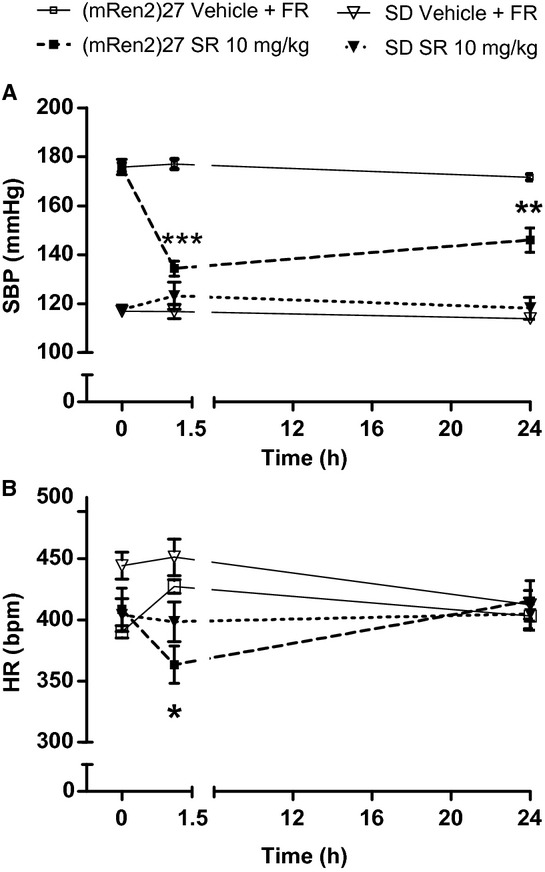
Acute systemic administration of CB_1_ receptor antagonist SR141716A lowers SBP (A) and transiently lowers HR (B) in (mRen2)27 rats but has no effect in SD rats. *P < 0.05 versus baseline; **P < 0.01; ***P < 0.001; *n* = 5 all groups. SR refers to SR141716A; FR = food restriction.

There were no significant differences in baseline SBP, HR (Fig. 1A and 1B) or body weight (Table 1) between the vehicle and drug treatment groups of (mRen2)27 or SD rats. In (mRen2)27 rats, p.o. injection of SR141716A (*n* = 5) lowered SBP by approximately 24%, from 176 ± 3 mmHg at baseline to 134 ± 3 mmHg after 90 min (*P* < 0.001; Fig. 1A). SBP remained lower 24 h after SR141716A administration, with evidence of partial recovery to 146 ± 5 mmHg (*P* < 0.01 vs. baseline; Fig. 1A). SR141716A in (mRen2)27 rats also caused a transient reduction in HR, which fell from 409 ± 17 bpm at baseline to 363 ± 15 bpm after 90 min (*P* < 0.05), but fully recovered within 24 h (Fig. 1B). Furthermore, (mRen2)27 rats treated with SR14171A consumed a similar amount of food overnight as those treated with vehicle and restricted to 13 g of food, which produced similar decreases in body weight in both groups (*P* < 0.01 vs. respective baseline body weights; Table 1). However, (mRen2)27 rats treated with SR141716A excreted significantly less urine overnight compared to those that received vehicle + FR (8 ± 1 vs. 14 ± 1 mL; *P* < 0.05), despite consuming similar amounts of water (Table 1). Vehicle did not significantly alter SBP or HR in (mRen2)27 rats after 90 min, nor did overnight FR change SBP or HR in (mRen2)27 rats 24 h after receiving vehicle (Fig. 1A and B).

**Table 1.** Effects of acute systemic SR141716A or vehicle + overnight FR treatment in (mRen2)27 and SD rats

Parameter	SD Vehicle +FR	SD SR 10 mg/kg	(mRen2)27 Vehicle +FR	(mRen2)27 SR 10 mg/kg
Baseline body weight (g)	388 ± 12	406 ± 22	555 ± 19	555 ± 30
Body weight at 24 h (g)	379 ± 12**	394 ± 19*	534 ± 16**	530 ± 27**
ΔBody weight (g)	−9 ± 1	−12 ± 3	−21 ± 4	−25 ± 5
Food intake (g)	13	12 ± 1	13	13 ± 2
Water intake (mL)	25 ± 3	22 ± 1	28 ± 1	22 ± 5
Urine volume (mL)	11 ± 2	6 ± 1	14 ± 1	8 ± 1#
Plasma Ang I (pg/mL)	240 ± 25	236 ± 28	139 ± 7	163 ± 19
Plasma Ang II (pg/mL)	66 ± 9	86 ± 29	102 ± 36	113 ± 43
Plasma Ang‐(1–7) (pg/mL)	58 ± 9	43 ± 9	67 ± 10	58 ± 12
Serum ACE (ng/mL)	35 ± 8	31 ± 10	23 ± 2	24 ± 1
Serum insulin (ng/mL)	2.8 ± 0.4	3.5 ± 0.7	2.9 ± 0.4	2.7 ± 0.3
Serum leptin (ng/mL)	4.2 ± 1.1	3.4 ± 0.5	5.9 ± 1.0	7.1 ± 1.5

Values are mean ± SEM. SR refers to SR141716A; FR = food restriction.

*P < 0.05 versus baseline; **P < 0.01 versus baseline; ^#^*P* < 0.05 versus (mRen2)27 Vehicle + FR treatment; *n* = 5 all groups.

In contrast to (mRen2)27 rats, acute administration of SR141716A in SD rats did not significantly change SBP or HR after 90 min or 24 h (Fig. 1A and 1B). As in (mRen2)27 rats, SD rats treated with SR141716A consumed similar quantities of food and water overnight as those who received vehicle + FR, producing similar changes in body weight (*P* < 0.05 vs. respective baseline body weights; Table 1). Overnight water intake was similar between SD groups, but there was a trend for lower urine excretion in SR141716A‐treated SD rats compared to vehicle + FR‐treated rats (6 ± 1 vs. 11 ± 2 mL; *P* = 0.08; Table 1). As with the SR141716A‐treated group, SBP, and HR were unaffected at 90 min and 24 h in SD rats treated with vehicle + FR (Fig. 1). There were no differences in circulating levels of RAS peptides or insulin or leptin between treatment groups in (mRen2)27 or SD rats (Table 1).

### Chronic systemic CB_1_ receptor blockade in (mRen2)27 rats

#### Body weight and fat mass

**Figure 2.**
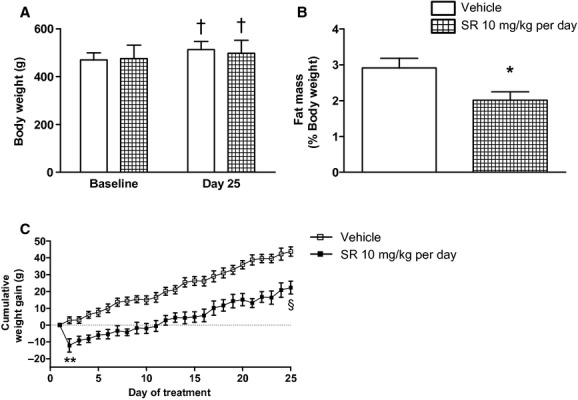
(mRen2)27 rats treated with chronic CB_1_ receptor blockade maintained similar raw body weight (A), but had significantly lower fat mass (B) and gained significantly less weight over the treatment period (C). Fat mass reflects the sum of white adipose tissue collected after animals were sacrificed on Day 28 and expressed as percent of body weight. ^†^*P* < 0.001 versus respective baseline values; *P < 0.05 versus Vehicle group; **P < 0.01; ^§^*P* < 0.0001; *n* = 6–8.

There were no baseline differences in body weight between (mRen2)27 rats in the vehicle (470 ± 11 g; *n* = 8) and SR141716A (476 ± 21 g; *n* = 7) treatment groups. By Day 25 of treatment body weights in both groups had significantly increased relative to their Day 1 baselines, to 513 ± 12 g (*P* < 0.001) in vehicle‐treated rats and 498 ± 21 g (*P* < 0.001) in drug‐treated rats (Fig. 2A). Since the total weight gained over the period of the study by each group was <10% of total body weight, raw body weight values on Day 25 did not significantly differ between treatment groups. However, rats treated with SR141716A had approximately 31% lower fat composition, measured as the mass of white adipose tissue as a percentage of body weight, after Day 28 than rats treated with vehicle over the duration of the study (*P* < 0.05; Fig. 2B), suggesting differences in body composition between groups. Furthermore, SR141716A‐treated rats on average gained approximately half as much weight as rats treated with vehicle through Day 25 of treatment (44 ± 3 g vs. 22 ± 3 g; *P* < 0.001; Fig. 2C). Drug‐treated rats lost approximately 12 g of body weight overnight after the Day 1 dose of SR141716A (Fig. 2C) but thereafter steadily gained weight through Day 25 of treatment. Further statistical analysis of the weight gain curves starting on Day 2 and not including the initial weight loss revealed a significant interaction between treatment groups with respect to cumulative daily weight gain over the treatment period (*P* < 0.01), indicating a lower rate of weight gain over the duration of treatment after the initial overnight weight loss in rats that received SR141716A (regression slopes after Day 1 = 1.82 ± 0.07 Vehicle vs. 1.29 ± 0.09 SR141716A; *P* < 0.0001).

#### Food and water intake, and urine excretion

**Figure 3.**
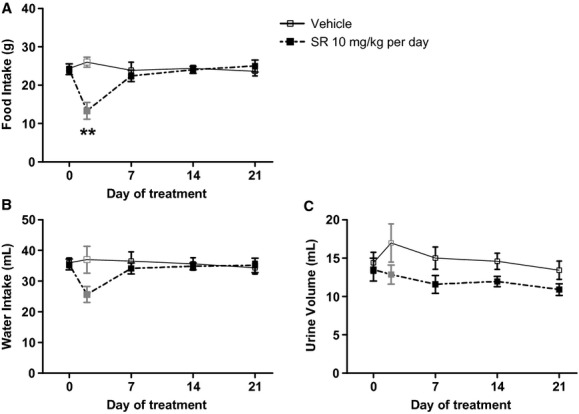
Chronic systemic CB_1_ receptor blockade produced a transient reduction in food intake (A) without sustained changes, but not in water intake (B) or urine volume (C) in (mRen2)27 rats. **P < 0.01 versus Vehicle on Day 2; *n* = 7–8 on Days 0, 7, 14 and 21; *n* = 3–4 on Day 2.

There were no differences between vehicle and SR141716A treatment groups in food intake, water intake, or urine volume at baseline. Over the duration of treatment there were no sustained differences in food intake between treatment groups; however, the weight loss exhibited after Day 1 by (mRen2)27 rats treated with SR141716A was associated with a transient reduction in food intake measured in subgroups of animals (13 ± 2 g food drug‐treated vs. 26 ± 1 g food vehicle‐treated on Day 2; *n* = 3–4; *P* < 0.01) that fully recovered by Day 7 of treatment (Fig. 3A), in agreement with previous reports (Ravinet Trillou et al. 2003; Poirier et al. 2005). There were no significant transient or sustained differences in water intake between treatment groups (Fig. 3B). Urine volume trended lower in rats treated with SR141716A over the duration of treatment compared to vehicle‐treated rats, but there was not a significant treatment effect (Fig. 3C).

#### Blood pressure, HR, and RAS components

**Figure 4.**
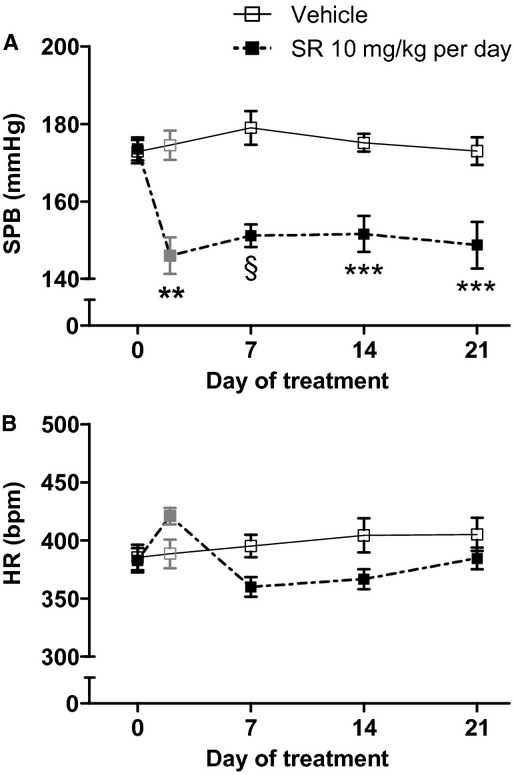
Chronic systemic CB_1_ receptor blockade in (mRen2)27 rats lowered SBP (A) without significantly changing HR (B) over the duration of treatment. **P < 0.01 versus Vehicle; ***P < 0.001; ^§^*P* < 0.0001; *n* = 7–8 on Days 0, 7, 14, and 21; *n* = 3–4 on Day 2. Day 2 values reflect measurements made 24 h following the Day 1 treatment.

There were no baseline differences in SBP or HR between treatment groups. On Day 7, SBP in SR141716A‐treated (mRen2)27 rats was significantly reduced from a baseline of 174 ± 3 mmHg to a new value of 151 ± 3 (*P* < 0.01) and remained lower through Day 21 of treatment (149 ± 6 mmHg; *P* < 0.01 vs. baseline; Fig. 4A). Measurements conducted in a subgroup of rats (*n* = 3) on Day 2 of treatment indicated that SBP was reduced within 24 h after receiving the Day 1 injection of SR141716A (146 ± 5 mmHg; *P* < 0.05 vs. baseline; Fig. 4A). There was no effect of vehicle on SBP, nor was there a significant treatment effect on HR, over the duration of treatment (Fig. 4A and B). Furthermore, no differences between treatment groups were found in levels of the RAS components Ang I, Ang II, Ang‐(1–7), or ACE analyzed from blood collected after animals were sacrificed on Day 28 of the study (Table 2). Residual effects of isoflurane anesthesia, well known to stimulate release of renin (Udelsman et al. 1987), and accompanying surgical stress from 48 h prior to blood collection may explain of the higher Ang I in these animals than in the acute studies (Table 1).

**Table 2.** Effects of chronic systemic SR141716A or vehicle treatment in (mRen2)27 rats

Parameter	(mRen2)27 Vehicle	(mRen2)27 SR 10 mg/kg/day
Plasma Ang I (pg/mL)	353 ± 112	302 ± 52
Plasma Ang II (pg/mL)	71 ± 25	69 ± 11
Plasma Ang‐(1–7) (pg/mL)	45 ± 7	46 ± 7
Serum ACE (ng/mL)	26 ± 2	30 ± 4
Urine vasopressin (pg/mL)		
Baseline	107 ± 12	104 ± 16
Day 21	100 ± 19	110 ± 31
Urine osmolality (mOsm/L)		
Baseline	1471 ± 107	1528 ± 77
Day 21	1639 ± 110	1809 ± 69**

Values are mean ± SEM. SR refers to SR141716A.

**P < 0.01 versus Baseline; *n* = 7–8 all groups.

#### Leptin, insulin, blood glucose, urine vasopressin, and osmolality

**Figure 5.**
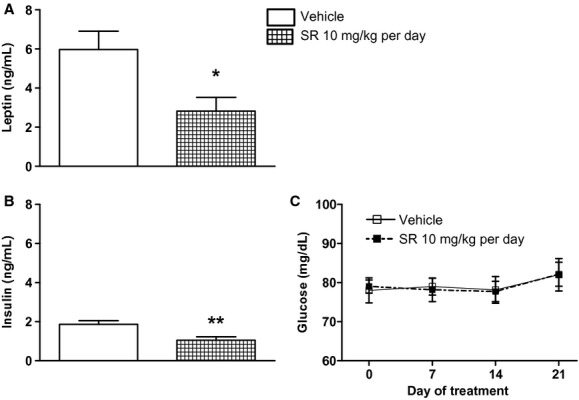
Following 28‐day chronic systemic CB_1_ receptor blockade (mRen2)27 rats had lower serum leptin (A) and insulin (B) levels than rats treated with vehicle, while blood glucose (C) was unaffected over the duration of treatment. *P < 0.05 versus Vehicle; **P < 0.01; *n* = 7–8.

At the end of the study, serum collected from (mRen2)27 rats that received chronic treatment with SR141716A had significantly less leptin (*P* < 0.05; Fig. 5A) and insulin (*P* < 0.05; Fig. 5B) compared to rats treated with vehicle. There was no effect on blood glucose over the duration of the study in either group (Fig. 5C). Furthermore, no differences in urine vasopressin levels were found between treatment groups (Table 2). Urine osmolality increased in both treatment groups between baseline and Day 21 of the study, reaching statistical significance in SR141716A‐treated rats (*P* < 0.01), but there was no effect of treatment (Table 2).

#### Conscious baroreflex function, HRV, and BPV

**Figure 6.**
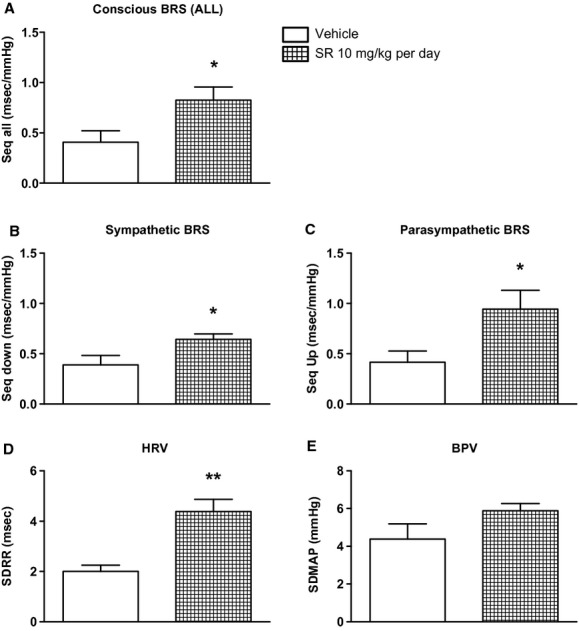
Chronic systemic CB_1_ receptor blockade for 28 days via SR141716A in (mRen2)27 rats improved conscious sympathetic and parasympathetic BRS for control of HR (A–C) and HRV (D) compared to vehicle‐treated rats, without significantly changing BPV (E). *P < 0.05 versus Vehicle; **P < 0.01; *n* = 5.

Conscious AP was recorded from (mRen2)27 rats treated with vehicle (*n* = 5) or SR141716A (*n* = 5) on Day 28 of the study for spectral analysis of the AP and corresponding HR waves to calculate indices of sympathovagal function. Rats that received chronic systemic treatment with SR141716A displayed a twofold greater value in overall conscious spontaneous baroreflex function in the time domain (Seq All; 0.82 ± 0.13 vs. 0.41 ± 0.11 ms/mmHg; *P* < 0.05; Fig. 6A), with significantly greater sympathetic (Seq Down; 0.64 ± 0.05 vs. 0.39 ± 0.09 ms/mmHg; *P* < 0.05; Fig. 6B) and parasympathetic (Seq Up; 0.94 ± 0.19 vs. 0.42 ± 0.11 ms/mmHg; *P* < 0.05; Fig. 6C) BRS for control of HR compared to rats treated with vehicle. There was a similar trend for improvement in the BRS analyzed in the frequency domain (HF*α*; 0.34 ± 0.14 Vehicle vs. 0.74 ± 0.20 ms/mmHg SR141716A; *P* = 0.09), but no difference in LF*α* (0.29 ± 0.08 Vehicle vs. 0.38 ± 0.09 ms/mmHg SR141716A; *P* > 0.05).

In addition, HRV, a measure of cardiac vagal tone, was significantly higher in SR141716A‐treated rats relative to vehicle‐treated rats (4.38 ± 0.49 vs. 2.0 ± 0.26 ms; *P* < 0.01; Fig. 6D). There was not a significant difference in BPV, an index of vascular sympathetic tone, between treatment groups in the time domain (Fig. 6E) or in the frequency domain measured as LF‐SAP (0.43 ± 0.14 Vehicle; *n* = 3 vs. 0.64 ± 0.06 mmHg SR141716A; *n* = 5; *P* > 0.05).

## Discussion

In this study, we report for the first time effects of acute and chronic systemic blockade of CB_1_ cannabinoid receptors on blood pressure, body weight, fat mass, feeding, serum leptin and insulin content, and conscious baroreflex function in transgenic hypertensive rats with upregulated RAS activity. Acute oral administration of the CB_1_‐selective antagonist SR141716A significantly lowers SBP in (mRen2)27 rats but has no effect in normotensive SD control rats. The effect is immediate, occurring within 90 min of administration, accompanied by bradycardia and sustained for 24 h. The reduction in HR may contribute to the initial reduction in SBP, but the transient hypophagic effect of SR141716A alone does not significantly contribute because overnight FR did not change SBP in (mRen2)27 rats treated with the vehicle. Chronic daily treatment with SR141716A maintained the SBP‐lowering effect at a similar level over the duration of the study without significantly changing HR. Acute and chronic effects of systemic CB_1_ receptor blockade by SR141716A are not associated with differences in levels of circulating RAS components, or urinary vasopressin compared to vehicle treatment, suggesting that changes in blood pressure may be principally attributed to significantly improved sympathovagal baroreflex function and heart rate variability observed in drug‐treated rats after 28 days. The beneficial hemodynamic effects of chronic systemic SR141716A treatment are accompanied by reductions in cumulative weight gain over the treatment period, reduced fat mass as a percentage of body weight, and lower leptin and insulin levels in (mRen2)27 rats after the 4‐week study. Taken together, data from our current study support our hypothesis that systemic blockade of central and peripheral CB_1_ receptors has direct beneficial effects on blood pressure with a concomitant positive influence over metabolic profile in transgenic (mRen2)27 rats with Ang II‐dependent hypertension and features of metabolic syndrome.

The metabolic effects of chronic systemic SR141716A administration in (mRen2)27 rats are consistent with the reported literature in humans and animals with metabolic syndrome. In obese Zucker rats, chronic treatment with SR141716A dose‐dependently reduces weight gain, fat mass, and improves insulin sensitivity (Vickers et al. 2003; Janiak et al. 2007; Lindborg et al. 2011). Similar effects of SR141716A accompanied by an improved serum lipid profile are found in diet‐induced obese mice (Poirier et al. 2005), and in obese and diabetic patients (Van Gaal et al. 2005). However, it is unclear whether the reduced insulin levels in our drug‐treated rats signify improvement in insulin sensitivity or are a direct cause or result of the decreased weight gain and fat mass associated with SR141716A treatment.

The reduced weight gain and fat mass in (mRen2)27 rats treated with SR141716A cannot be attributed to sustained reductions in food consumption because feeding was only transiently reduced and fully recovered within 7 days, in line with previous descriptions of the hypophagic effect of SR141716A (Vickers et al. 2003; Janiak et al. 2007). A significant statistical interaction between the vehicle and drug treatment groups over the entire treatment period further precludes differences in weight gain and fat mass from being wholly attributed to overnight weight loss associated with hypophagia experienced by SR141716A‐treated rats after Day 1. Several alternative mechanisms may contribute to the beneficial metabolic actions of CB_1_ blockade. For instance, obesity is associated with defective leptin signaling and hyperleptinemia (Maffei et al. 1995), both of which are exhibited by (mRen2)27 rats (Kasper et al. 2005) and may contribute to increased body weight and fat mass in this strain. Defective central leptin signaling is also associated with enhanced endocannabinoid tone independent of obesity (Di Marzo et al. 2001). Animals that received SR141716A in our study had significantly lower serum leptin levels, perhaps resulting from lower body fat after chronic treatment.

In addition to leptin abnormalities, metabolic syndrome is associated with depressed plasma adiponectin levels (Arita et al. 1999) and dyslipidemia (Nicholas 1999). Adiponectin released from adipocytes induces fatty acid *β*‐oxidation and increases lipoprotein lipase activity (Diez and Iglesias 2003) with central regulatory effects on energy expenditure that complement brain actions of leptin (Nedvidkova et al. 2005). Adipocyte CB_1_ receptors directly regulate plasma levels of adiponectin (Perwitz et al. 2006), and SR141716A increases release of adiponectin in obese humans (Despres et al. 2005), Zucker rats (Janiak et al. 2007), and diet‐induced obese mice (Tam et al. 2014). Similarly, dyslipidemia in the form of elevated triglycerides is reported in (mRen2)27 rats and may contribute to defective insulin signaling in this strain (Sloniger et al. 2005a,b). Hepatocytes express CB_1_ receptors that promote fatty acid synthesis when activated (Osei‐Hyiaman et al. 2005). Moreover, insulin resistance associated with a high‐fat diet is linked to hepatic CB_1_ receptor‐mediated synthesis of long‐chain ceramides (Cinar et al. 2014). All of these effects are reversed or attenuated by SR141716A (Osei‐Hyiaman et al. 2005) or a peripherally restricted CB_1_ antagonist (Cinar et al. 2014), as was low‐density lipoprotein (LDL)/high‐density lipoprotein cholesterol ratio by SR141716A in diet‐induced obese mice (Poirier et al. 2005). Therefore, it is likely that SR141716A in our study exerted some of its metabolic effects through direct actions in adipocytes or in the liver.

A similar physiological provenance of metabolic disturbance in (mRen2)27 rats and other animal models of metabolic syndrome, likely involving upregulated endocannabinoid tone, may be inferred because of the shared effect profile of CB_1_ receptor blockade among these different models. Dysfunctional RAS signaling may be another shared mechanism of metabolic disturbance, as chronic blockade of AT_1_ receptors lowers AP and improves the principal symptoms of metabolic syndrome in obese humans (Bramlage et al. 2008), Zucker rats (Toblli et al. 2008), spontaneously hypertensive rats (SHR) made obese by high‐fat diet (Muller‐Fielitz et al. 2014), and (mRen2)27 rats (Sloniger et al. 2005a,b). In fact, mounting evidence suggests the RAS and the endocannabinoid system may work in tandem to promote cardiometabolic diseases through signaling interactions that augment the pathogenic effects of AT_1_ or CB_1_ receptor activation. For example, chronic treatment with SR141716A significantly reduces Ang II‐mediated fibrosis in mouse liver (Rozenfeld et al. 2011) and the enhanced pressor response to intravenous Ang II exhibited by Zucker rats (Janiak et al. 2007). Chronic SR141716A treatment also reduces vascular expression of AT_1_ receptors in apolipoprotein E‐deficient mice (Tiyerili et al. 2010).

A compelling description of potential heteromerization mechanisms between CB_1_ and AT_1_ receptors accompanying reduced fibrosis in mouse liver (Rozenfeld et al. 2011) led us to hypothesize that CB_1_ receptor blockade would disrupt the Ang II‐dependent hypertension in (mRen2)27 rats, and results from our current study are congruent with this hypothesis. Orally administered SR141716A reduced SBP in (mRen2)27 rats within 90 min by ≈43 mmHg to a level 17 mmHg (~15%) higher than baseline SBP of SD rats, whose SBP was unaltered by SR141716A. The fast onset of the effect precludes changes in overnight food intake or the long‐term metabolic effects of SR141716A from contributing to the reduction in SBP in the acute setting, suggesting the effect on SBP is a direct consequence of CB_1_ antagonism. However, whether blockade of CB_1_ receptors directly interfered with vascular or central Ang II‐AT_1_ receptor signaling, or reduced SBP through an alternative mechanism cannot be determined from our study. Chronic CB_1_ receptor blockade in Zucker rats is associated with reduced acute pressor responses to exogenously administered Ang II (Janiak et al. 2007), suggesting interference with vascular responses. However, even acute increases in pressure with Ang II involve brain AT_1_ receptors (Northcott et al. 2010). Thus, direct acute interference of AT_1_ receptor signaling by CB_1_ blockade may have both central and peripherally consequences.

Spectral analysis methods for measuring indices of blood pressure regulation and autonomic tone at the conclusion of the chronic study reveal significant improvement in both sympathetic (Seq Down, Seq All) and parasympathetic (Seq Up, Seq All) spontaneous BRS for control of HR in SR141716A‐treated conscious rats compared to those that received vehicle. Similar to the conscious spontaneous BRS measurements, HRV was significantly higher in drug‐treated compared to vehicle‐treated rats, indicating increased resting vagal tone. In contrast, we did not detect differences in the modulation of sympathetic nervous system activity for control of blood pressure between groups as assessed by BPV or LF‐SAP. CB_1_ receptor stimulation inhibits release of norepinephrine from vascular nerve terminals (Pakdeechote et al. 2007), which with direct vasodilation (Wagner et al. 1999) may be expected to offset actions of Ang II to both activate the sympathetic nervous system centrally and to cause local vasocontriction. Although we did not observe an increase in pressure with CB_1_ receptor blockade, the failure to observe a decrease in BPV or LF‐SAP may result from the fact that reduced sympathetic outflow is negated by increased release of norepinephrine locally within the vasculature. Whether there is an additional contribution to the decrease in pressure in the long‐term setting of vascular AT_1_ receptor down‐regulation (Tiyerili et al. 2010) as discussed above remains to be determined. Regardless, modulation of central blood pressure regulation likely contributes to the lower SBP during chronic treatment with SR141716A in (mRen2)27 rats, and implicates direct central effects of systemic SR141716A in these animals. Interestingly, Ang II‐mediated release of endocannabinoids contributes to the pressor actions of the peptide in the paraventricular nucleus of the hypothalamus (Gyombolai et al. 2012). Thus, the potential for specific brainstem or hypothalamic sites of action for chronic systemic SR141716A treatment exists and must be elucidated in the future.

In the cardiovascular system, endocannabinoids mediate vasodilation and cardiomyocyte relaxation through CB_1_ receptor‐specific mechanisms (Wagner et al. 1999), yielding a hypotensive effect that is enhanced in both acute hypertension and in the chronic SHR model (Lake et al. 1997; Ho and Gardiner 2009). Indeed, intravenous anandamide normalized while SR141716A further increased AP in anesthetized SHR (Batkai et al. 2004), suggesting that upregulated CB_1_ receptor tone in this model is likely compensatory rather than pathogenic in nature. However, chronic CB_1_ blockade did not alter pressure in obese‐prone SHR (Slavic et al. 2013), and only modestly reduced SBP of human patients with obesity‐related hypertension after chronic treatment (Grassi et al. 2008; Ruilope et al. 2008), an effect not independent of weight loss. Furthermore, there are conflicting reports as to whether SR141716A corrects modestly elevated, sympathetically driven SBP in Zucker rats (Janiak et al. 2007; Mingorance et al. 2009), which are a model of leptin receptor deficiency. These reports are further confounded by recent evidence that at least some metabolic effects of SR141716A are mediated by increased sympathetic activation (Bellocchio et al. 2013). Therefore, specific phenotypic characteristics including severity of the hypertension and whether accompanied by metabolic dysfunction should be considered carefully when interpreting the blood pressure effects of systemic cannabinoids. The (mRen2)27 rat is a unique model in which to study the hemodynamic and metabolic effects of the endocannabinoid system in a setting of upregulated RAS activity accompanied by many of the features of metabolic syndrome.

Although SBP remained suppressed at a consistent level over the chronic treatment period, it is certainly possible that more slowly manifesting metabolic effects, such as the reduction in fat mass, or decreased insulin or leptin, may contribute to lower SBP in the later stages of treatment. Leptin and insulin are both known to activate the sympathetic nervous system and impair BRS (McKernan and Calaresu 1996; Arnold et al. 2009). While reductions of these hormones are not associated with reductions in indices of vascular sympathetic drive (BPV, LF‐SAP), lower levels of leptin or insulin may contribute to the enhanced autonomic control during chronic treatment with SR141716A. In contrast, reduced HR during acute treatment with SR141716A may drive lowered blood pressure given that no differences were found in circulating insulin, leptin, or RAS components after 24 h. Even a RAS‐mediated change in SBP could conceivably be an indirect consequence of chronic CB_1_ blockade because correction of hypercholesterolemia may downregulate AT_1_ receptor expression, which is correlated with plasma LDL levels in humans and animals (Nickenig et al. 1997). Thus, reduced responses to endogenous Ang II may occur even though circulating levels of the peptide did not change.

The results of our current study, obtained after acute and chronic systemic treatment with SR141716A in conscious hypertensive (mRen2)27 rats, illustrate immediate and sustained beneficial effects on the cardiovascular and metabolic systems. Further study will be needed to elucidate the precise mechanisms of these blood pressure responses in acute and chronic settings and to separate direct and indirect effects of CB_1_ receptor blockade on blood pressure in this strain. However, the findings contrast with the interpretation of short‐term beneficial effects of CB_1_ receptor blockade (4–14 days) on blood pressure and metabolic profile, previously attributed to decreased food consumption and other transient actions (Thornton‐Jones et al. 2006). Moreover, they contrast with acute studies in other models of hypertension without substantial metabolic dysfunction where CB_1_ receptor blockade increased blood pressure (Batkai et al. 2004). Therefore, our study showcases the importance of studying the long‐term consequences of blockade of the CB_1_ receptor in various models of hypertension and also highlights the challenges of treating diseases as multifarious as hypertension.

## Perspectives and Significance

Our findings support a permissive role for the endocannabinoid system in the maintenance of hypertension and metabolic syndrome in (mRen2)27 rats, consistent with emerging evidence for common signaling pathways between the endocannabinoid system and the RAS that may promote or enhance Ang II‐related pathologies. It remains unknown to what extent central actions of SR141716A contribute to the metabolic or blood pressure effects of the chronic treatment, because distinct central and peripheral mechanisms of cardiometabolic regulation are reported in the endocannabinoid literature (Cota et al. 2003; Silvestri et al. 2011). In (mRen2)27 rats with a centrally mediated Ang II component to the hypertension, medullary levels of endocannabinoids are elevated and CB_1_ receptor blockade in nucleus of the solitary tract improves BRS (Schaich et al. 2013). Thus, central nervous system interactions between the two systems may contribute to the overall improvement in cardiometabolic function in our study. Therefore, it would be interesting to study peripherally restricted CB_1_ antagonists in a similar experimental paradigm to distinguish peripheral from central effects of CB_1_ blockade in (mRen2)27 rats. Given that RAS blockers exhibit beneficial effects on metabolism in addition to their antihypertensive actions and exert a positive influence on affect (Pavel et al. 2008), we speculate that combination CB_1_‐AT_1_ receptor blockade might be additive or synergistic with potentially mitigated side effects of both treatments for management of metabolic syndrome considering the RAS‐dependent component associated with most forms of human hypertension.

## Acknowledgments

We thank Katie Atkins and Pam Dean in the Hypertension Core Assay Laboratory, and Ellen Tommasi for technical assistance.

## Conflict of Interest

None declared.
